# High-Risk Aortic Plaque in Atrial Fibrillation: A Therapeutic Dilemma

**DOI:** 10.7759/cureus.53913

**Published:** 2024-02-09

**Authors:** Shaniza Haniff, Ashwin Shive Gowda, Nawfal Al-khafaji, Asher Gorantla

**Affiliations:** 1 Internal Medicine, University at Buffalo, New York, USA; 2 Neurology, Royal Stoke University Hospital, Stoke-on-Trent, GBR; 3 Cardiology, Trinity Medical Cardiology, Buffalo, USA; 4 Internal Medicine, State University of New York (SUNY) Downstate Health Sciences University (HSU), Brooklyn, USA

**Keywords:** direct-acting oral anticoagulants, vitamin k-antagonists, cardiovascular management, thromboembolic event, anticoagulation therapy, antithrombotic therapy, aortic plaque, atrial fibrillation (af)

## Abstract

Atrial fibrillation (AF), a common cardiac arrhythmia, is often accompanied by aortic plaques that are associated with an increased risk of embolic events, including stroke. Evidence-based management in this population is lacking. We present a case of a 77-year-old female with new-onset AF who was found to have a high-risk aortic plaque at the level of the ascending aorta and ostium of the right coronary artery. Definitive treatment for AF, cardioversion, high-risk aortic plaque, and cardiothoracic surgery, could not be performed due to the elevated risk of ischemic stroke and embolic complications. Based on existing literature, the cardiologist and cardiothoracic surgeon collaboratively decided to treat both conditions with anticoagulation, statin, and periodic imaging surveillance of high-risk aortic plaque. The patient was successfully managed without any thromboembolic complications despite an elevated risk. This case report provides a comprehensive literature review of managing AF with high-risk aortic plaques. It delves into the integration of anticoagulation and antiplatelet agents in the dual challenge of stroke prevention in AF and mitigating embolic risks associated with aortic plaques. To date, there has been no consensus on managing AF and high-risk aortic plaques; thus, we aim to fill this gap.

## Introduction

Atrial fibrillation (AF) is a prevalent cardiac arrhythmia associated with a five-fold increased risk of thromboembolic events, particularly ischemic strokes, due to the formation of intracardiac blood clots [1]. When AF is coupled with the presence of aortic plaque, the thromboembolic risk is further augmented [2].

Aortic plaques, atherosclerotic deposits within the aorta, were first discovered in 1990 by transesophageal echocardiogram in patients evaluated for embolic ischemic stroke of unknown origin [3]. Soon after the discovery, the association between aortic plaque and ischemic stroke, transient ischemic attack, and embolization to visceral and peripheral arterial circulation was established [4]. High-risk features of aortic plaques such as plaque size greater than or equal to 4mm and morphological characteristics- ulcerations or superimposed thrombi- are associated with an increased risk of embolization [5,6]. Studies found that embolic complications occurred in 20-33% of patients with mobile aortic plaques in just one year [3,7], and there was an associated 12% increased risk of recurrent strokes [3,6].

Both aortic plaques and AF are independent risk factors for thromboembolic events related to cardiovascular and cerebrovascular complications [2]. In 2021, 1 in 6 deaths from cardiovascular disease was due to stroke, leading to significant morbidity and mortality [8]. Antithrombotic therapy, antiplatelet and anticoagulation, is commonly used in conditions with high-risk thromboembolic events and guided by evidence-based research. To date, evidence-based research and management are lacking regarding antithrombotic therapy in patients with AF and high-risk aortic plaques [3]. 

We present a case of high-risk aortic plaque in a patient with AF and review the literature and clinical considerations regarding antithrombotic strategies for mitigating thromboembolic risk in patients with high-risk aortic plaque and AF.

## Case presentation

A 77-year-old female presented to the cardiology clinic for newly diagnosed AF. She was hospitalized ten days ago and treated for COVID-19 infection, urinary tract infection, and new-onset atrial fibrillation with a rapid ventricular rate likely triggered by infection. Her discharge laboratory tests showed an unremarkable complete blood count, basic metabolic profile, and thyroid function test. Her fasting cholesterol was 168, low-density lipoprotein was 82, and elevated triglycerides were 169. She had a normal left ventricular systolic function with an ejection fraction of 55-60% and a left atrial volume of 56 milliliters, without valvular or structural heart abnormalities on the transthoracic echocardiogram obtained during hospitalization.

She did have a past medical history of transient ischemic attack, hypertension, dyslipidemia, and hypothyroidism, and her calculated CHA2DS2-Vasc score was 6, indicating an 8-9% risk of stroke or transient ischemic attack per year without being on oral anticoagulation. She was subsequently discharged on a high-intensity statin (atorvastatin 80 mg daily), rate-control medications (diltiazem 180 mg daily and metoprolol succinate 100 mg daily), and an oral anticoagulant (apixaban 5 mg twice daily).

**Figure 1:**
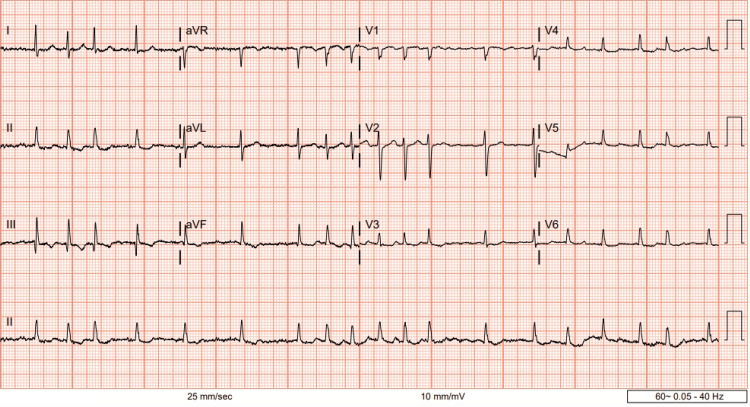
Electrocardiogram showing atrial fibrillation with a rapid ventricular rate of 115 beats per minute

At the cardiology clinic visit, her electrocardiogram showed AF of 115 beats per minute (bpm) (Figure 1). However, she denied cardiac symptoms- palpitations, shortness of breath, chest pain, or neurological symptoms- focal numbness, weakness, slurred speech, and vision changes. Her HAS-BLED score was 2, indicating a moderate risk for major bleeding on anticoagulation per 100 patient-years. However, she denied any significant bleeding, ecchymosis, or bruising since being on apixaban. Her diltiazem dose was subsequently increased to 240 mg daily due to her uncontrolled paroxysmal AF.

At a three-month follow-up visit, she complained of a decreased appetite and an unintentional weight loss of 20 pounds. Her vitals showed an elevated heart rate of 105 bpm and low normal blood pressure of 100/60 mmHg. A repeat echo showed a mildly reduced ejection fraction of 50-55% compared to the prior 55-60%. Due to her symptoms, mildly reduced ejection fraction and soft blood pressure, which limited further increase of rate control medications, rhythm control was discussed. After an informed discussion of the all-cause mortality benefit of rhythm control in atrial fibrillation, the patient agreed to pursue an elective transesophageal echocardiogram (TEE) and direct cardioversion.

**Figure 2:**
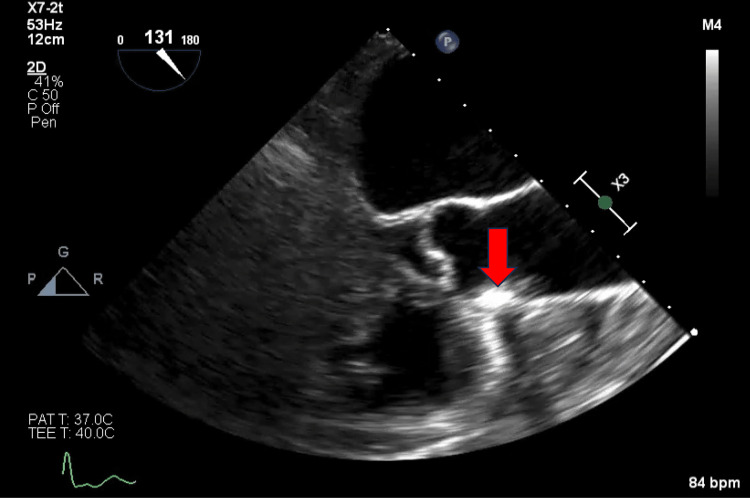
Transesophageal echocardiogram showing mobile echo density (indicated by a red arrow) distal to the aortic valve on the wall of the ascending thoracic aorta

On the day of her elective procedure, the TEE revealed a mobile echo density distal to the aortic valve at the ascending part of the thoracic aorta, suspicious for an ulcerated atherosclerotic plaque (Figure 2). Her cardiac systolic function was estimated as normal, and no evidence of thrombus was seen in the left atrium or appendage. Her direct cardioversion was subsequently canceled due to concerns about the high risk of thromboembolism. She subsequently underwent computed tomography (CT) with and without contrast of the chest to confirm the presence of high-risk aortic plaque on the TEE.

**Figure 3:**
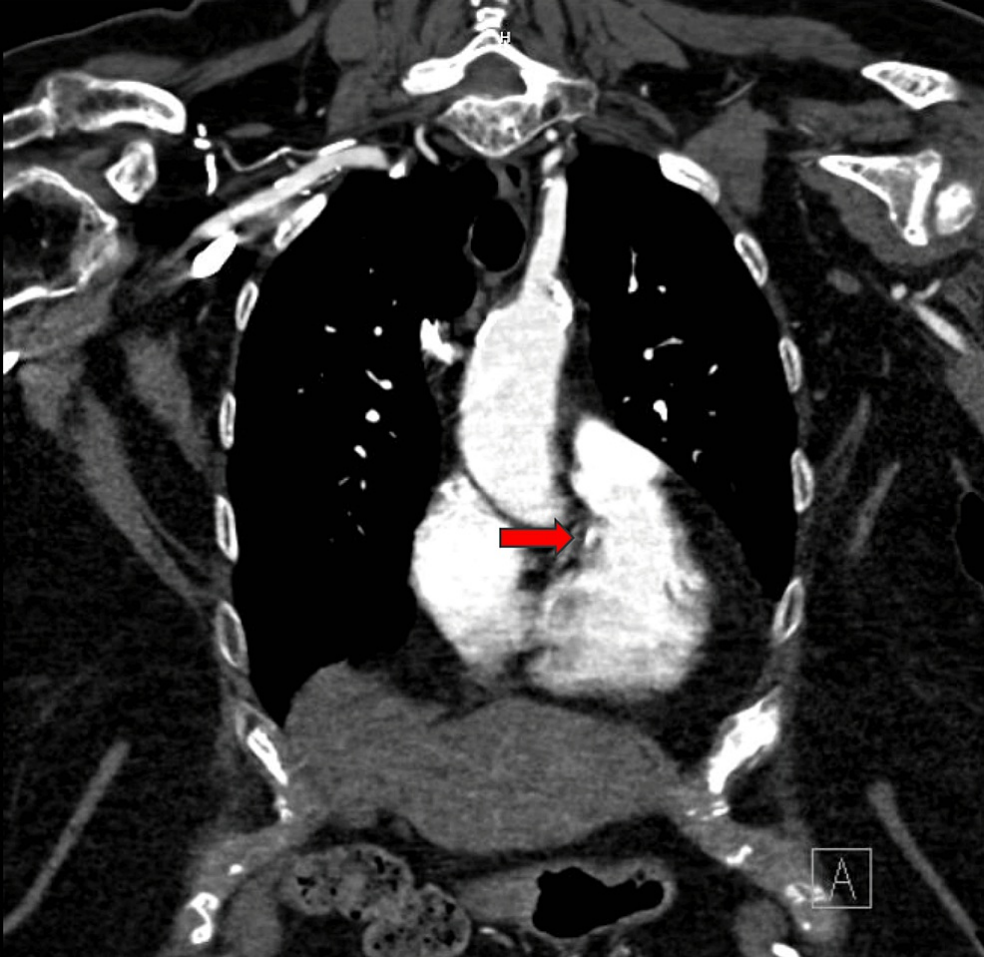
Computed tomography (CT) of the chest with contrast in the coronal plane showing a focal calcified lesion adjacent to the ostia of the right coronary artery at the root of the ascending aorta

**Figure 4:**
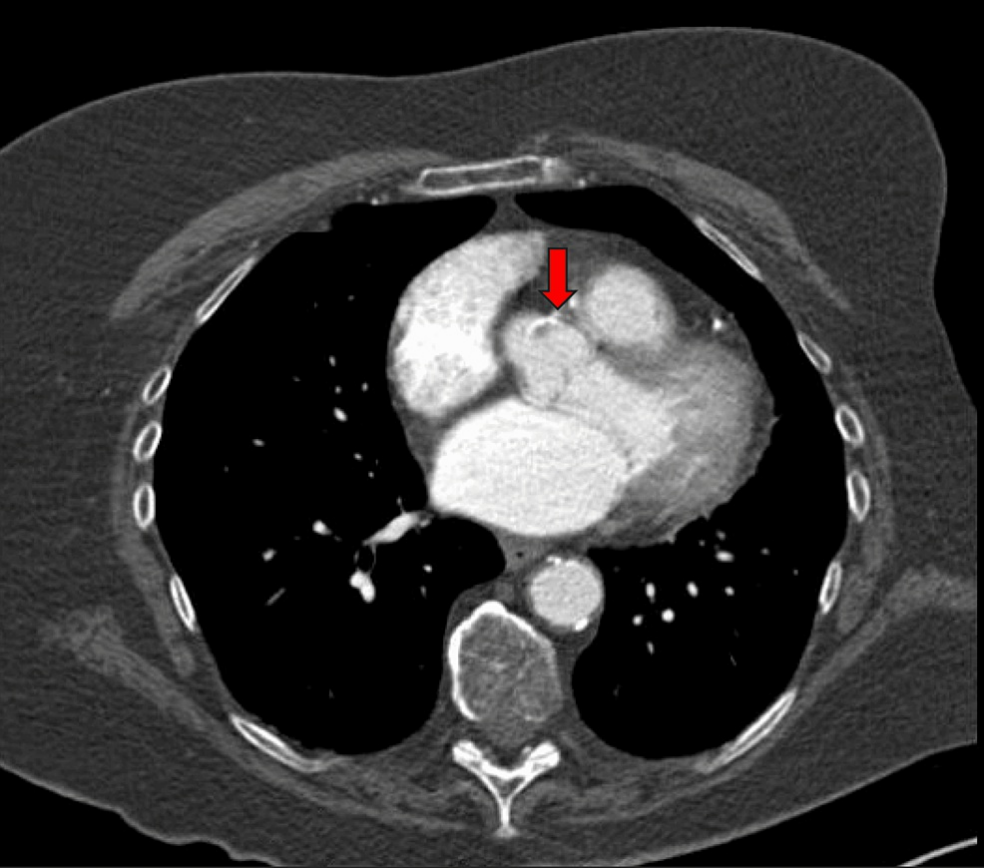
Computed tomography (CT) of the chest with contrast in axial view showing mixed-density atherosclerotic plaque at the origin of the right coronary ostium

The CT scan of the chest showed a focal calcified and noncalcified lesion adjacent to the ostia of the right coronary artery at the root of the aorta, as well as noncalcified and calcified plaque protruding into the aortic lumen, confirming a high-risk ulcerated atherosclerotic plaque in the ascending aorta (Figures 3, 4).

The patient was referred to a cardiothoracic surgeon to discuss possible surgical open atherectomy and thrombectomy via median sternotomy. However, given the focal area of aortic plaque, there was an increased risk for embolization and stroke. Additionally, a heavily calcified aortic arch would make any surgical intervention a high risk for neurological complications. Similarly, the coronary angiogram was not recommended. After being informed about the risks and benefits of surgery, the patient decided to pursue medical management and periodic six-month surveillance imaging. 

While there are no current medical management guidelines on high-risk aortic plaques in patients with AF, a collaborative decision was made between the patient, cardiologist, and cardiothoracic surgeon to continue anticoagulation (apixaban 5 mg twice daily), antiplatelet (aspirin 81 mg daily), and a high-intensity statin (atorvastatin 40 mg daily) to stabilize the plaque and prevent plaque hemorrhage and thromboembolism. At a one-year follow-up visit, the patient remained free of cerebral, cardiac, and peripheral thromboembolism and denied any episodes of major bleeding on the anticoagulation. A repeat echocardiogram showed a preserved ejection fraction with unchanged findings compared to the previous, and the chest CT angiogram showed no progression of the aortic plaque compared to initial scans. 

## Discussion

High-risk aortic plaque and AF co-exist and have an estimated prevalence of 8.3-25% [9,10], predisposing to high risks of thromboembolism. To date, evidence-based management for specific drug therapies in this population is lacking. Commonly used therapies include oral anticoagulants with or without antiplatelets, high-intensity statin, and blood pressure control [11]. This discussion reviews the existing literature on medical and surgical management in patients with AF and high-risk aortic plaques, which remains controversial.

Medical management 

Aortic Arch-Related Cerebral Hazard (ARCH) Trial

The ARCH trial was the first and, to date, the only prospective randomized trial set to compare the efficacy and tolerance of dual antiplatelet therapy (aspirin and clopidogrel) versus anticoagulation with warfarin in patients with atherothrombosis of the aortic arch and a recent (less than six months) cerebral or peripheral embolic event [12]. Embolic events were reduced with DAPT by 7.6% compared to 11.3% of warfarin-treated patients (P=0.2) [12,13]. Unfortunately, this trial was terminated early due to a lack of funding, resulting in statistically insignificant results that cannot be extrapolated to guide treatment [13].

Stroke Prevention in Atrial Fibrillation (SPAF) III Study Trial

The SPAF III trial, a randomized trial, recruited patients with atrial fibrillation and high-risk features for thromboembolism, such as aortic plaques documented on TEE. Patients received either adjusted-dose warfarin (INR 2.0 to 3.0) or combined low-dose warfarin (1 to 3 mg, INR 1.2 to 1.5) and aspirin daily. This trial was prematurely terminated due to the superior benefit of adjusted-dose warfarin compared to combined low-dose warfarin and aspirin daily in preventing thromboembolism, particularly in patients with high-risk aortic plaques [14]. The risk of ischemic stroke in patients with high-risk aortic plaque treated with low-dose warfarin plus aspirin was 15.8% compared to only 4% of patients treated with adjusted-dose warfarin, indicating a 75% risk reduction [14].

Elderly patients with AF and high-risk aortic plaque were found to have a reduction of thromboembolism when treated with adjusted-dose warfarin [2]. However, it is uncertain whether adjusted-dose warfarin decreased thromboembolism due to a reduction in aortic plaque or atrial fibrillation-related thrombi as the origin of thromboembolism events in this study could not be determined [15]. Additionally, patients with AF and high-risk aortic plaque who received adjusted-dose warfarin remained at increased risk for thromboembolism (4.0% per year [CI, 1.3% to 12% per year]), bringing into question more effective treatment [16].

The Role of Antiplatelet Therapy

Conversely, in the Patent Foramen Ovale in Cryptogenic Stroke Study (PICSS), adjusted-dose warfarin therapy versus aspirin only showed no benefit of ischemic stroke prevention among the non-AF population with high-risk aortic plaques [14]. Moreover, the French Study of Aortic Plaques in Stroke Group showed a significantly increased risk of ischemic stroke recurrence in patients with high-risk aortic plaques when treated with aspirin only [12]. In contrast, the European Society of Cardiology does recommend a single-agent aspirin for patients with high-risk aortic plaques in non-AF patients; for those already on anticoagulation for AF, aspirin and anticoagulation can be used if the thrombotic risk is high [17].

The Role of Warfarin

While the SPAF III trial showed a benefit of adjusted-dose warfarin in ischemic stroke reduction, newer experimental animal models and clinical trials have demonstrated that warfarin accelerates aortic atherosclerosis, increases plaque inflammation and calcification, and leads to plaque progression [18,19]. Nishiga et al. (2013) showed a higher incidence of embolic events in patients with high-risk aortic plaques treated with warfarin [20]. The reason for plaque progression is warfarin’s inhibition of vitamin K-dependent inhibitory proteins responsible for arterial calcification, causing cellular senescence and vascular fibrosis [21]. This effect is particularly pronounced in patients younger than sixty-five than those older than sixty-five treated with warfarin [21,22].

Additionally, warfarin increases the risk of intracranial bleeding events compared to newer non-vitamin K anticoagulants. However, this is not to say that warfarin would not benefit patients with aortic plaque and coexisting atrial fibrillation. This highlights the need to balance the use of this drug with patient age, comorbidities, and bleeding risk [21].

The Role of Non-vitamin K Antagonist Direct Oral Anticoagulants

Non-vitamin K antagonist direct oral anticoagulants (apixaban, rivaroxaban, dabigatran, edoxaban) do not interact with vitamin K and thus have a favorable aortic atherosclerosis profile. Through direct thrombin inhibition, dabigatran reduces the progression and calcification of aortic atherosclerosis [18]. Edoxaban, another oral anticoagulant, by a different mechanism of inhibiting factor Xa, may have clinical significance as it reduces inflammation and proliferation of smooth muscles in the vessel wall and decreases vascular fibrosis compared to placebo and warfarin [19-21]. Similarly, apixaban has been found to stabilize atherosclerotic processes [21]. While non-vitamin K antagonist direct oral anticoagulants are commonly used for their protective all-cause mortality in the prevention of thromboembolic events in patients with AF, clinical studies have yet to assess their efficacy with co-existing in high-risk aortic plaques [21].

*Surgery*

It remains unclear whether aortic arch atherectomy is necessary to prevent thromboembolism in patients with aortic plaque. In one study, aortic arch atherectomy was associated with a significantly elevated perioperative stroke rate: 34.9% (among 268 patients) compared to 11.6% who did not undergo prophylactic surgery [3]. These patients had clinically significant strokes, more extended hospital stays, and higher mortality rates. Conversely, aortic arch atherectomy did show favorable results among younger healthy patients with recurrent systemic emboli refractory to medical optimization [23]. 

## Conclusions

The management of AF and high-risk aortic plaque requires a comprehensive and multidisciplinary approach. Antithrombotics, lipid-lowering therapy, and blood pressure management seem promising in reducing thromboembolism, including ischemic strokes, and stabilizing plaque progression. While surgical intervention may be beneficial, it is associated with high perioperative stroke rates and, therefore, should be carefully considered. Close collaboration between cardiologists and cardiothoracic surgeons is essential to tailor interventions based on physician and patient preferences. As research advances, personalized treatment strategies will further enhance the effectiveness of this high-risk population, ultimately improving the quality of life for affected individuals.
